# Supplementary material for: Content-rich biological network constructed by mining PubMed abstracts
Source: BMC Bioinformatics. 2004 Oct 8;5:147. doi: 10.1186/1471-2105-5-147 (PMC528731; doi:10.1186/1471-2105-5-147)
Supplement: Additional File 2 — The original results of the above study (non-essential files are deleted to keep the file size under the limit set by BMC bioinformatics). [file 1471-2105-5-147-S2.bz2 › chilibotAdditionalFile2/dip20/257ID8242751E1020/html/CCND1_CDKN1A.html]

 


 **CCND1** and **CDKN1A** 
  
Found 633 abstracts in PubMed, retrieved 20.  
 

 What does Google say? 
 PDF only 
| .edu only 

---

**Interactive relationship** (e.g. stimulation, inhibition, etc)

**Inhibitory relationship**- Moreover, Rb phosphorylation, which lies immediately downstream of the cyclin D1  [ **CCND1** ]  Cdk4 p21WAF1 Cip1  [ **CDKN1A** ]  complex, was blocked either by Sp1 overexpression or antisense p21WAF1 Cip1  [ **CDKN1A** ] .  Ref: 12796485 J Biol Chem< MedlineTA>J Biol Chem,
**Stimulatory relationship**- In p53 null or p21 WAF1  [ **CDKN1A** ]  null cells marked apoptosis occurred after SN38 alone, and was additionally enhanced by Bcl  [ **CCND1** ]  xl knockdown in p21 WAF1  [ **CDKN1A** ]  null cells but not in p53 null cells.  Ref: 12855666 Clin Cancer Res, 2003
**Neutral relationship**

**Non-interactive relationship** (e.g. studied together, co-existance, homology, etc.)

- In our study 86 tonsil cancers were analysed for HPV status by sequence analysis of polymerase chain reaction products and for the expression of cell cycle proteins p53, p21 CIP1  [ **CDKN1A** ]  WAF1  [ **CDKN1A** ] , pRb, p16 INK4A, cyclin D1  [ **CCND1** ]  and p27 KIP1 by immunohistochemistry.  Ref: 12845651 Int J Cancer, 2003
- These events were accompanied by down regulation of the Raf 1 MEK ERK pathway as well as diminished expression of Bcr Abl and cyclin D1  [ **CCND1** ] , cleavage of p21 CIP1  [ **CDKN1A** ]  and pRb, and induction of the stress related kinases JNK and p38 MAPK.  Ref: 12893773 BloodBlood, 2003
- Abnormalities in G1 S transition in cell cultures have been attributed to alterations in ErbB erythroblastic leukaemia viral v erb b oncogene homologue, avian signalling, cyclin D1  [ **CCND1** ]  overexpression or disturbance of the p21 WAF1  [ **CDKN1A** ]  p21 mediated cell cycle arrest induced by p53.  Ref: 12825853 Breast Cancer Res Treat, 2003
- SN38 induced p53, Bax, Bcl  [ **CCND1** ]  xl, and p53 dependent p21 WAF1  [ **CDKN1A** ]  protein accumulation.  Ref: 12855666 Clin Cancer Res, 2003
- Changes in the expression of caspase 3, bax, bcl  [ **CCND1** ]  2 and p21cip waf  [ **CDKN1A** ]  were assessed by quantitative Western blotting.  Ref: 12888913 Int J Oncol, 2003
- Sp1 blocked assembly of cyclin D1  [ **CCND1** ]  Cdk4 p21WAF1 Cip1  [ **CDKN1A** ]  complex formation whose integrity is critical for G1 > ; S transition.  Ref: 12796485 J Biol Chem< MedlineTA>J Biol Chem, 2003
- Most thymomas and thymic carcinomas were negative for Bcl  [ **CCND1** ]  XL, p21WAF, 1 CIP1  [ **CDKN1A** ]  and cyclin D1  [ **CCND1** ] .  Ref: 12877732 Histopathology, 2003
- Cyclin D1  [ **CCND1** ]  up regulation and p21 WAF1  [ **CDKN1A** ]  CIP1  [ **CDKN1A** ]  down regulation occur early in UC related carcinogenesis.  Ref: 12827612 Hum Pathol, 2003
- Experimental Design Using wild type HCT116, p53 null, Bax null, or p21 WAF1  [ **CDKN1A** ]  null isogenic derivatives, we measured expression of regulators of cellular response, and associated growth arrests or apoptosis, after SN38 treatment, with or without antisense mediated Bcl  [ **CCND1** ]  xl knockdown.  Ref: 12855666 Clin Cancer Res, 2003
- AIMS To correlate the expression of a series of apoptotic and oncogene markers including p53, Bcl  [ **CCND1** ]  2, BAX, Bcl  [ **CCND1** ]  XL, p21WAF, 1 CIP1  [ **CDKN1A** ] , cyclin D1  [ **CCND1** ] , HER 2 neu in thymic epithelial tumours with histological type, stage and resectability and to determine whether the information on HER 2 neu would be valuable in identifying patients who are eligible for anti HER 2 neu treatment.  Ref: 12877732 Histopathology, 2003
- Sp1 Inhibits proliferation and induces apoptosis in vascular smooth muscle cells by repressing p21WAF1 Cip1  [ **CDKN1A** ]  transcription and cyclin D1  [ **CCND1** ]  Cdk4 p21WAF1 Cip1  [ **CDKN1A** ]  complex formation.  Ref: 12796485 J Biol Chem< MedlineTA>J Biol Chem, 2003
- In addition, the latter induces changes in gene expression by up regulating p53, Waf 1  [ **CDKN1A** ] , Cas, Dap kinase and caspases and 14 and down regulating Bcl  [ **CCND1** ]  2.  Ref: 12887065 Biol Chem, 2003
- On the other hand, prolonged ERK activity was observed with a subsequent increase of cyclin D1  [ **CCND1** ]  and p21 Cip  [ **CDKN1A** ]  WAF1  [ **CDKN1A** ]  upon treating with 100 micro M of ZnCl2 in MSSM, and this was associated with non proliferation.  Ref: 12816758 Am J Physiol Gastrointest Liver Physiol< MedlineTA>Am J Physiol Gastrointest Liver Physiol, 2003
- The combination effectively up regulated caspase 3 and bax and down regulated bcl  [ **CCND1** ]  2 and p21cip waf  [ **CDKN1A** ] .  Ref: 12888913 Int J Oncol, 2003
- We have shown that abolition of p21 WAF1  [ **CDKN1A** ]  mediated drug induced senescence or antisense mediated Bcl  [ **CCND1** ]  xl knockdown can both, independently, enhance the apoptotic response of colorectal cancer cells to SN38 in vitro.  Ref: 12855666 Clin Cancer Res, 2003
- p XSC and p XSeSG were significantly more effective in the upregulation of pro apoptotic genes, such as p21 CIP1  [ **CDKN1A** ]  WAF1  [ **CDKN1A** ] , p27 KIP1, APO 1, and Caspase 3, while downregulating cell growth regulatory genes, such as c myc, cyclin D1  [ **CCND1** ] , cyclin D2 and proliferating cell nuclear antigen PCNA.  Ref: 12844480 Carcinogenesis< MedlineTA>Carcinogenesis, 2003
- It is unclear whether and how cyclin D1  [ **CCND1** ]  and or p21 WAF1  [ **CDKN1A** ]  CIP1  [ **CDKN1A** ]  dysregulation contribute to ulcerative colitis UC related inflammation and colorectal carcinogenesis.  Ref: 12827612 Hum Pathol, 2003
- QUC showed cyclin D1  [ **CCND1** ]  negative weak staining and p21 WAF1  [ **CDKN1A** ]  CIP1  [ **CDKN1A** ]  surface epithelial and upper third crypt staining expression similar to that of normal colorectum.  Ref: 12827612 Hum Pathol, 2003
- These events culminated in activation of the caspase cascade, manifested by poly ADP ribose polymerase, p21 CIP1  [ **CDKN1A** ]  WAF1  [ **CDKN1A** ] , p27 KIP, Bcl  [ **CCND1** ]  2, and retinoblastoma protein degradation.  Ref: 12839953 Cancer Res, 2003
- The differential regulations of cell growth, ERK activities, and cyclin D1  [ **CCND1** ]  and p21 Cip  [ **CDKN1A** ]  WAF1  [ **CDKN1A** ]  inductions were also observed in serum enriched medium containing higher zinc concentrations.  Ref: 12816758 Am J Physiol Gastrointest Liver Physiol< MedlineTA>Am J Physiol Gastrointest Liver Physiol, 2003
- Moreover, this ERK activation and cyclin D1  [ **CCND1** ]  and p21 Cip  [ **CDKN1A** ]  WAF1  [ **CDKN1A** ]  induction were abolished by PD98059 pre treatment.  Ref: 12816758 Am J Physiol Gastrointest Liver Physiol< MedlineTA>Am J Physiol Gastrointest Liver Physiol, 2003
- n = 11 were studied with cyclin D1  [ **CCND1** ]  and p21 WAF1  [ **CDKN1A** ]  CIP1  [ **CDKN1A** ]  immunohistochemistry.  Ref: 12827612 Hum Pathol, 2003
- In conclusion, AUC shows up regulated cyclin D1  [ **CCND1** ]  and p21 WAF1  [ **CDKN1A** ]  CIP1  [ **CDKN1A** ]  expression.  Ref: 12827612 Hum Pathol, 2003
